# Nurses’ Perception of Patient Safety Culture in a Referral Hospital: A Cross-Sectional Study

**DOI:** 10.3390/ijerph191610131

**Published:** 2022-08-16

**Authors:** Eva María Sosa-Palanca, Carlos Saus-Ortega, Vicente Gea-Caballero, Joaquín Andani-Cervera, Pedro García-Martínez, Rafael Manuel Ortí-Lucas

**Affiliations:** 1 PhD School, Catholic University of Valencia San Vicente Mártir, 46001 Valencia, Spain; 2 Research Group GREIACC, Health Research Institute La Fe, 46026 Valencia, Spain; 3 Nursing School La Fe, Adscript Center of Universidad de Valencia, 46026 Valencia, Spain; 4 Faculty of Health Science, International University of Valencia, 46002 Valencia, Spain; 5 Faculty of Medicine and Health Sciences, Catholic University of Valencia San Vicente Mártir, 46001 Valencia, Spain; 6 Research Group on Public Health and Patient Safety, Catholic University of Valencia San Vicente Mártir, 46001 Valencia, Spain; 7 Department of Preventive Medicine, Hospital Clínico Universitario de Valencia, 46010 Valencia, Spain

**Keywords:** patient safety, safety culture, nurses, adverse events

## Abstract

Healthcare systems are becoming increasingly complex which is helping to promote a ‘culture of safety’ within them based on the best scientific evidence available. Indeed, creating a positive institutional culture of patient safety is reflected in health outcomes. The aim of this present study was to describe the perception of culture of safety by nurses in adult inpatient units in a tertiary hospital and to analyze adverse events reporting. It was a cross-sectional study in which 202 nurses from adult hospitalization units of the Hospital Universitario y Politécnico La Fe in Valencia (Spain) participated. The perception of safety culture was measured using the Hospital Survey on Patient Safety questionnaire version 1.0, which consists of 42 items distributed in 12 dimensions that are considered strengths or weaknesses. In addition, adverse events related to nursing care during the study period and those reported in the official hospital registry were collected. Finally, the association between safety culture and sociodemographic and labor variables was explored. A total of 148 responses to the questionnaire were analyzed (Cronbach’s alpha = 0.94), where seven dimensions and 25 items were identified as weaknesses. Two hundred and fourteen events were identified and none were reported in the official registry. Years of experience were significantly (*p* < 0.05) associated with safety culture. It is necessary to establish strategies to improve the perception of the safety culture of nurses, as well as to make nurses aware of the importance of notifying adverse events derived from health care.

## 1. Introduction

Healthcare systems are becoming increasingly complex which is helping to promote a ‘culture of safety’ (CS) within them based on the best scientific evidence available. According to the Agency for Healthcare Research and Quality (AHRQ), CS is defined as *the product of individual and group values that determine the way an organization acts in terms of the management of patient safety* (PS) [1]. The World Health Organization (WHO) defines PS *as a reduction of the risk of harm associated with healthcare to an acceptable minimum* [2], which it considers one of the fundamental pillars of healthcare quality. The creation of the Patient Safety Alliance in 2004, during the 57th WHO assembly in Geneva, further serves to highlight the importance placed on PS. This alliance was designed with the aim of coordinating, disseminating, and improving the development of policies and practices related to PS in every country [3].

To improve PS, the National Quality Forum (2003) included the achievement of an adequate PS culture among its recommendations to healthcare organizations [4]. Indeed, creating a positive institutional culture of PS is reflected in health outcomes [5,6]. To achieve this, managers and professionals must be involved in reporting adverse events (AEs), learning from mistakes, and redesigning processes to prevent them from happening again. The absence of a CS can lead to routines that trigger incorrect or risky habits that should be avoided by promoting safe practices and continuously evaluating their effectiveness [7]. Validated and reliable questionnaires that analyze the perception of professionals of the safety climate at their place of work in healthcare organizations and that implement protocols for improvement are available [8]. The dimensions that are generally assessed are teamwork, AE reporting, communication, and the respondents’ overall perception of safety [4,9].

The National Study of Hospitalization-Related Adverse Events (ENEAS in its Spanish acronym) [10] showed that the incidence of patients suffering healthcare-related AEs in Spain was 9.3%. Of note, this represents an increase in hospital costs equivalent to 6.7% of total healthcare expenditure [6]. In fact, a later study [11] conducted in 24 hospitals revealed an incidence of 8.4%. In the United States, more than 250,000 patients each year suffer an AE and around 100,000 patients die as a result of healthcare interventions, with an annual incidence of AEs of 10% [12]. At the European level, similar rates of AEs of 8–10% of reported incidents of harm have been recorded in the UK [13,14]. Similarly, a rate of 10.3% and an estimated mean increase in hospital stay of 6.1 days have recently been published for Ireland [15].

One of the areas of work proposed by the European Commission in 2014 was the encouragement of AE reporting as a tool to disseminate a PS culture, maintenance of regular updates, and dissemination of recommendations for the implementation and operation of an incident reporting system for learning purposes [16]. Importantly, nurses spend the most time in direct contact with patients and are therefore key to improving quality of care and promoting PS [17]. Indeed, the WHO considers these professionals to be “the backbone of the health system” [18]. Thus, it is useful to understand nurses’ perception, knowledge, and involvement in the CS so that protocols and improvement plans can be implemented in healthcare organizations to increase AE reporting and reduce their occurrence.

The objectives of this study were to describe the perception of safety culture of nursing staff in adult inpatient units of a tertiary hospital using the HSOPS questionnaire [19] and to assess adverse event reporting activity by nurses.

## 2. Materials and Methods

### 2.1. Design

This was an observational, descriptive, cross-sectional study following the STROBE guidelines for cross-sectional studies [20]. The participants were nurses (N = 202) in the adult hospitalization units at the Hospital Universitario y Politécnico La Fe de Valencia (Spain) were surveyed between February and March 2020. Ten hospitalization units were selected on the basis of homogeneity criteria: 3 different medical specialties, three surgical specialties, and four combined medical–surgical units. We calculated that a minimum sample of 133 participants would be required to achieve a confidence level of 95% and an alpha error of 5%.

### 2.2. Selection Criteria

The inclusion criteria were nurses in adult inpatient units in active duty at the time of data collection (working, on holiday, on leave or on temporary disability). The exclusion criteria were nurses with temporary employment contracts lasting less than three months, leaving more than five items incomplete on the survey, and failure to sign the informed consent form.

### 2.3. Study Variables

The version 1.0, validated in Spanish, of the Hospital Survey on Patient Safety (HSOPS) PS questionnaire from the Agency for Health Care Research and Quality (AHRQ) was used as the measurement instrument [19] (Appendix A). The HSOPS measures the perception of a CS using 42 items grouped into 12 dimensions. The items are measured using Likert-type response options ranging from 1 (strongly disagree or never) to 5 (strongly agree or always). The questionnaire also contains several complementary information items, mainly designed to describe the sample demographic (years of experience in the profession, years worked at the hospital, years worked in the department, hours worked per week, overall assessment of safety, and type of unit). The internal consistency of the dimensions showed a Cronbach alpha of 0.64–0.88.

The 12 dimensions (Ds) assessed by the questionnaire are:

D1: frequency of reported events;

D2: perception of safety;

D3: supervisor/manager expectations and actions promoting patient safety;

D4: organizational learning/continuous improvement;

D5: teamwork in the unit/service;

D6: openness in communication;

D7: feedback and communication on errors;

D8: non-punitive responses to errors;

D9: staffing;

D10: hospital management support for patient safety;

D11: inter-unit teamwork;

D12: problems with shift changes and transitions between services/units.

To record AEs, an ad hoc data collection notebook (DRC in its Spanish acronym) was constructed. Data were collected regarding the unit, persons responsible for filling in the data, AEs that occurred, and the type of event (Appendix B). Data were also collected on the number and types of AEs related to nursing care reported in the official register at the center (SINEA in its Spanish acronym).

### 2.4. Procedure

Unit supervisors were asked to deliver the questionnaires and ensure that they were subsequently collected anonymously. Supervisors and collaborating nurses from each unit were trained on the data collection procedure for two 2-hour sessions in February 2020. After the training, they collected the number and type of AE’s occurring as a result of the nursing activity. Finally adverse events reported in the official register of the center (SINEA) were obtained.

### 2.5. Data Analysis

A descriptive analysis of the variables was carried out. Frequencies and percentages or mean and standard deviations were used according to the nature of each variable.

In order to describe the nurses’ perception of safety culture, the following was carried out a descriptive analysis of the HSOPS questionnaire for each dimension and for each item.

The analysis of the dimensions was calculated using the following formula:

(Total number of positive responses on the items of a given dimension)/(Total number of responses on the items of the dimension).

Items and dimensions were classified as “strength” if:

A percentage ≥75% positive responses (agree/strongly agree or almost always/always) to questions asked in the positive.

A percentage ≥75% negative responses (disagree/strongly disagree or never/rarely) to questions asked in the negative.

Items and dimensions were classified as “weakness” if:

A percentage ≥50% negative responses (disagree/strongly disagree or rarely/never) to questions asked in the positive.

A percentage ≥50% positive responses (agree/strongly agree or almost always/always) to questions asked in the negative.

The association of the items and dimensions with the unit type variable was then explored using chi-square. The variables years of experience in the profession, years worked in the hospital, years worked in the department, hours worked per week, overall assessment of safety, were analyzed using Mann–Whitney U test.

Finally, to evaluate the reporting activity of AE, the total cases of AE occurred in the hospital and by type of unit (medical, surgical or mixed); and the number and type of AE reported in the official register of the center (SINEA) were presented.

Data analysis was performed with SPSS version 21 statistical software (IBM Corp., Armonk, NY, USA), applying a significance level of *p* < 0.05.

### 2.6. Ethical Considerations

This study complied with European and Spanish data protection regulations (Organic Law 3/2008) and was approved by the Ethics Committee, Research Committee, and Management at the Hospital Universitario y Politécnico La Fe de Valencia (2019/0266). Response to the questionnaire was voluntary and prior consent was obtained from the participants. The researchers did not present any ethical, moral, or economic conflicts of interest.

## 3. Results

### 3.1. Characterisation of the Study Sample

The characteristics of the study sample (N = 148) are presented in Table 1. Participants were 88% (n = 134) female, and 12% (n = 18) male. The distribution of participants according to unit type was 36.48% (n = 54) professionals from medical units, 25.67% (n = 38) from surgical units, and 37.83% (n = 56) from combined units. The 95.14% (n = 141) of the respondents had reported no AEs in the last year and 4.86% (n = 7) had reported between one and three events.

### 3.2. Perception of Safety Culture

Of the 202 questionnaires distributed, 152 were collected (response rate = 75.2%); four questionnaires were rejected because more than five items had not been completed, leaving a total of 148 for analysis (actual response rate = 73.2%). The overall internal consistency of the questionnaire in our setting was good (Cronbach alpha = 0.94); the consistency of the 9 dimensions of the questionnaire was also generally good, with only three (D9, D11, and D12) showing a Cronbach alpha <0.6.

Following the analysis criteria proposed by the AHRQ, no dimension was identified as a strength (Table 2). Only dimension five (teamwork in the unit/service) came close to the strength criteria, with 74.83% positive responses. However, seven dimensions were identified as weaknesses, with negative response rates exceeding 50%. These were D9 (staffing) and D10 (hospital management support for PS) with 75.92% and 73.42% negative responses, respectively. D8 (non-punitive response to errors) with 68.21% negative responses, D1 (frequency of reported events) with 62.99%, D7 (feedback and communication on errors) with 62.32%, D4 (organizational learning/continuous improvement) with 56.85%, and D2 (overall perception of safety) with 52.10% also constituted weaknesses or opportunities for improvement.

In the item-by-item analysis, issues related to teamwork, described as follows, “Staff support each other”, “In this unit we all treat each other with respect”, and “When someone is overloaded with work, they often find help from colleagues”, stood out as strengths. A total of 25 items were unfavorably rated. These items were identified as weaknesses or opportunities for improvement. The worst rated items were “Sometimes the best patient care cannot be provided because the working day is exhausting” and “We work under pressure to do too many things too quickly”. This was followed by “There are enough staff to cope with the workload” and “When a mistake is made, staff fear that it will be noted on their record”. The results of the analysis of all items on the questionnaire are presented in Table 2.

The bivariate analysis identified significant differences between the unit type for D1 (frequency of reported events), D5 (teamwork in the unit), D7 (feedback and communication about errors), D9 (staffing), D11 (inter-unit teamwork) and D12 (problems with shift changes and transitions between units) (Table 3). The results of the analysis by item and unit type showed differences in ten of the items (8, 14, 21, 22, 27, 31, 32, 35, 38 and 40) comprising the questionnaire (Table 3).

Mann–Whitney U analyses showed significant differences between D9 and years of experience in the profession (U = 1949.50, *p* = 0.048) and between D5 and D7 with years worked in the service (U = 1929.50, *p* = 0.012 and U = 2018.00, *p* = 0.034, respectively). Mann–Whitney U analysis also showed that items 1, 5, and 16 were associated with years of experience in the profession (U = 1976.00, *p* = 0.030; U = 1942.50, *p* = 0.027; and U = 1897.00, *p* = 0.012). Finally, items 1, 14, 16, 34, 38, and 39 were associated with years worked in the service (U = 1897.50, *p* = 0.003; U = 2078.00, *p* = 0.035; U = 1957.50, *p* = 0.009; U = 2024.50, *p* = 0.027; U = 1939.00, *p* = 0.010; and U = 1912.00, *p* = 0.009).

### 3.3. Adverse Event Reporting Activity by Nurses

#### 3.3.1. Adverse Events Occurring in Inpatient Units

A total of 214 AEs related to nursing practice were identified, including phlebitis associated with a peripheral venous catheter, extravasation of infusions in peripheral venous lines, pressure ulcers, accidental falls, and urinary tract infections associated with bladder catheterizations. The distribution of AEs is shown in Table 4.

#### 3.3.2. Adverse Events Reported in the Official Register

During the study period, no AEs related to nursing care were reported in the center’s official records and the AEs recorded in the twelve months prior were 1 fall and 2 pressure ulcers.

## 4. Discussion

This study aimed to describe the perception of the CS of nurses working in adult inpatient units in a tertiary hospital. The overall perception of safety was perceived as moderate with ample room for improvement, in agreement with other national studies [21,22,23]. The nurses surveyed perceived a good team relationship in their units, support among professionals, collaboration in times of care overload, and respectful treatment among team members.

We analyzed the dimensions concerning the frequency of reported events (management support and feedback and error reporting) based on an adaptation of the Westrum organizational development model [24] by Parker and Hudson [25] which was specifically designed to assess CS in organizations. Of the five phases or levels of organizational CS, the organization of our study population lay between the criteria of the first (pathological) phase and second (reactive) phase. According to the model authors and Manchester Patient Safety Framework (MaPSaF) [26], these phases correspond to organizations that have not yet acquired a value system that includes safety as a necessary element. These phases are generally based on poor, one-way communication in which staff can only talk to management after the incident has occurred and where feedback is restricted to those involved in the incident and focuses on blaming professionals.

This may be reflective of an organization where professionals are aware of the risks but are not always able to act on incidents because of under-reporting in official recording systems. As Ashcroft et al. suggest [27] organizations must continue moving away from a culture of blame, from treating mistakes as personal failures, and towards turning them into opportunities for improvement with a focus on learning and change for the better. Our findings regarding weaknesses and strengths are similar to the results of several studies [22,28,29,30,31] in which staffing, management support, non-punitive responses to errors, and the overall perception of safety were identified as weaknesses.

In six dimensions of the HSOPS questionnaire, significant differences were found depending on the type of unit. Professionals in combined units had a worse perception of the frequency of reported events, feedback and communication about errors than medical and surgical units, but had a better perception of staffing, teamwork, and problems with shift changes. Exploratory multicentric studies would be necessary to analyze or determine the causes of these differences.

At the national level, our results also coincided with those obtained by Merino [32,33], Gil [34], and other previous authors such as Hernández [21], Gama [35] and Saturno et al. [28] who analyzed the CS in various hospitals in the health system. They identified staffing, overall perception of safety, and management support as areas for improvement without detecting any dimensions as strengths. In 2011, Roqueta [23] and Skodová et al. [22] recorded similar results, obtaining the worst ratings in the same dimensions as the previously mentioned authors and similarly, without finding any strengths. Later, Mella Laborde [29] corroborated these findings and included the dimensions of staffing and management support as weaknesses without identifying any dimensions constituting strengths.

European studies also coincide with our findings regarding weaknesses [30,31,36,37,38], although some of them did identify the dimensions of the expectations and actions of the unit/service management or supervision, teamwork, or openness in communication as strengths [36,37]. The study carried out in 2014 in Croatia by Brborovic [31] recorded low scores for staffing and identified non-punitive responses to errors as the lowest rated dimension, attributing this result to ‘cultural guilt’ and the perception that errors should be punished—a conclusion also shared in the Italian study by Bagnasco [39] in 2011. International studies [1,39,40,41,42,43] showed very similar results to our own, identifying the same weaknesses and no strengths except, as in our study, for the teamwork dimension which came closest to the strength criteria. Other studies identified the same areas for improvement, but also identified strengths such as organizational learning/continuous improvement, teamwork, and management support [44,45].

In this study, nurses with more years of experience in the profession show a better perception of staffing, support among professionals and perceive the working day to be tiring. However, nurses with more years of experience in the unit have a better perception of teamwork and communication about errors. Additionally, nurses with more years of experience in the profession fear that mistakes mark their records and nurses with more years of experience in the unit are afraid to ask questions when something seems to have been done incorrectly. The latter finding could be related to the perception that superiors do not show interest in this aspect unless harm to the patient occurs.

The frequency of reported AEs was similar to other national and international work [1,22,28,36,40,41]. The perception of D1 was identified as an area for improvement in medical and mixed wards, while in surgical wards it was seen neither as a weakness or a strength. In this current study we did not explore the causes of these findings and so more specific studies are still required in this regard, qualitative studies are recommended in the future. Moreover, given the low number of reports, we could not establish any associations with reported AEs. Under reporting of AEs was clearly evident when contrasting the number of AEs detected with those reported. The association detected between the dimensions and the type of unit suggests a difference in the perception of the CS according to the type of patients they care for. We deduced that nurses in the medical-surgical units that integrate both types of patients had a better perception of PS culture than those in the medical or surgery units. In turn, the surgical units seemed to show a better perception of the frequency of reported events dimension than the medical and combined units.

The results obtained in this study showed a general low degree of perception of CS among nurses in adult hospital wards, suggesting that nurses’ perception of CS is low, as previously shown in national and international multicentric studies [4,28,46]. There was also evidence that large hospitals obtain worse results regarding the perception of CS among professionals. Nonetheless, it is worth noting that the data were collected during the initial period of the SARS-CoV-2 pandemic when healthcare professionals were working under a high degree of uncertainty.

We identified seven dimensions with low scores, meaning that there is a wide scope for improvement. Nevertheless, this will involve many challenges such as moving away from a culture of blame, employing new approaches to dealing with error, and reinforcing dimensions that tend to be strengths.

Limitations of this study included a small sample size, with only 202 nurses recruited through convenience sampling in a single hospital, a cross-sectional observational study design that prevented causality analysis. Dimensions D9, D11 and D12 of the HSOPS questionnaire had a Cronbach’s alpha <0.6. All this limited the external validity of the results found.

## 5. Conclusions

Our assessment showed that the nurses’ overall perception of safety was moderate. Seven dimensions of the HSOPS questionnaire were identified as areas for improvement and no dimension as a strength. Thus, our findings indicate the need to establish improvement strategies in the area of patient safety in adult inpatient units in order to strengthen the culture of safety.

It was detected that the nurses carry out an underreporting of the adverse events that occur in the units. Supervisors of hospitalization units need to place more emphasis on nurses so that they report AEs derived from health care. This requires raising awareness among nurses and promoting the use of notification and registration systems.

## Figures and Tables

**Table 1:** Socio-demographic variables.

Socio-Demographic Variables	Mean	SD	1st, 3rd Q
Years of experience in the profession	17.91	10.25	9.5, 24.5
Years worked in Hospital La FE	10	8.52	4, 14
Years worked in the service	7.6	7.42	3, 10
Working hours per month	36.89	5.51	37, 40
Overall safety assessment	6.14	1.42	5, 7

**Table 2:** Analysis of dimensions and items.

Dimensions and Items	%Positive Favorable Responses	%Neutral Responses	%Unfavorable Adverse Responses
**D1: Frequency of reported events (α de Cronbach 0.92)**	17.56	19.37	62.99
40. Errors that are discovered and corrected before they affect the patient are reported.	18.91	18.24	62.63
41. Errors that are not likely to harm the patient are reported.	12.18	22.3	64.87
42. Errors that have had no adverse consequences, but could foreseeably have harmed the patient, are reported.	20.94	17.57	61.48
**D2: Perception of safety (α de Cronbach 0.70)**	26.75	21.14	52.10
15. Never increase the pace of work if it means sacrificing patient safety.	19.86	8.22	71.91
18. Our procedures and systems are good at preventing errors from happening.	40.54	41.22	18.25
10. It is just by chance that more serious mistakes don’t happen around here.	16.89	10.81	72.3
17. We have patient safety problems in this unit.	29.73	24.32	45.95
**D3: Supervisor/Manager expectations and actions promoting patient safety (α de Cronbach 0.84)**	48.47	28.21	23.31
19. My supervisor/manager expresses satisfaction when we try to avoid patient safety risks.	47.97	26.35	25.68
20. My supervisor/manager seriously considers staff suggestions for improving patient safety.	47.97	22.3	29.73
21. When work pressure increases, my supervisor/manager wants us to work faster, even though patient safety may be put at risk.	50	31.08	18.92
22. My supervisor/manager overlooks patient safety problems that happen over and over.	47.97	33.11	18.92
**D4: Organizational learning/continuous improvement) (α de Cronbach 0.76)**	30.27	12.63	56.85
6. We have activities to improve patient safety.	27.02	12.16	60.81
9. When a failure in patient care is detected, appropriate measures are taken to prevent it from happening again.	46.24	8.16	44.9
13. After we make changes to improve patient safety, we evaluate their effectiveness.	17.57	17.57	64.86
**D5: Teamwork in the unit/service (α de Cronbach 0.84)**	74.83	11.65	13.51
1. Staff support each other.	77.03	7.43	15.54
3. When a lot of work needs to be done quickly, we work together as a team to get the work done.	68.92	12.16	18.92
4. In this unit we all treat each other with respect.	75.67	14.19	10.14
11. When someone is overloaded with work, they often find help from colleagues.	77.7	12.84	9.46
**D6: Communication openness (α de Cronbach 0.74)**	25	29.27	45.72
35. Staff will freely speak up if they see something that may negatively affect patient care.	27.71	24.32	47.98
37. Staff feels free to question the decisions or actions of those with more authority.	20.27	23.65	56.08
39. Staff are afraid to ask questions when something do not seem right.	27.03	39.86	33.11
**D7: Feedback and communication about error (α de Cronbach 0.81)**	18.03	19.65	62.32
34. When we report an incident, we are informed about what kind of action has been taken.	14.19	16.22	69.7
36. We are informed of errors occurring in this service/unit.	20.27	18.24	61.49
38. In my service/unit we discussed how to prevent errors.	19.73	24.49	55.78
**D8: Non-punitive response to errors (α de Cronbach 0.80)**	13.19	7.51	68.21
8. Staff feel like their mistakes are held against them.	33.33	7.48	59.18
12. When a fault is detected, before looking for the cause, they look for a “culprit”.	27.4	7.53	65.07
16. When a mistake is made, staff fear that it will be noted on their record.	12.17	7.43	80.4
**D9: Staffing (α de Cronbach 0.55)**	17.46	6.61	75.92
2. There are enough staff to cope with the workload.	12.16	4.73	83.11
5. Sometimes the best patient care cannot be provided because the working day is exhausting.	10.13	2.03	87.84
7. We use more agency/temporary staff than is best for patient care.	40.81	14.29	44.9
14. We work under pressure to do too many things too quickly.	6.77	5.41	87.84
**D10: Hospital management support for patient safety (α de Cronbach 0.88)**	9.68	16.89	73.42
23. Hospital management provides a work climate that promotes patient safety.	8.11	16.89	75
30. The actions of hospital management show that patient safety is a top priority.	10.81	20.27	68.92
31. Hospital management seems interested in patient safety only after an adverse event happens.	10.14	13.51	76.35
**D11: Inter-unit teamwork (α de Cronbach 0.59)**	45.61	23.31	31.08
26. There is good cooperation among hospital units that need to work together.	53.38	22.97	23.65
32. Hospital units work well together to provide the best care for patients.	67.57	18.92	13.52
24. Hospital units do not coordinate well with each other.	28.38	16.89	54.73
28. It is often unpleasant to work with staff from other hospital units.	33.11	34.46	32.43
**D12: Problems with shift changes and transitions between units (α de Cronbach 0.53)**	43.41	11.99	44.59
25. Patient information is partly lost when patients are transferred from one unit/service to another.	32.43	11.49	56.09
27. Important patient care information is often lost during shift changes.	44.6	4.73	50.67
29. Problems often occur in the exchange of information across hospital units.	47.3	23.65	29.05
33. Shift changes are problematic for patients in this hospital.	54.04	8.11	37.84

**Table 3:** Analysis by dimension, item, and unit type.

Dimensions and Items	Medical	Surgical	Combined	Chi Squared	*p*
**D1: Frequency of reported events**	73.65	88.33	65.94	6.617	**0.037**
40. Errors that are discovered and corrected before they affect the patient are reported	70.15	91.63	67.07	9.984	**0.007**
41. Errors that are not likely to harm the patient are reported	76.23	84.43	66.09	5.205	0.074
42. Errors that have had no adverse consequences, but could foreseeably have harmed the patient, are reported.	74.32	84.49	67.89	3.735	0.155
**D2: Perception of safety**	68.42	75.42	79.74	1.998	0.368
15. Never increase the pace of work if it means sacrificing patient safety	70.95	82.82	72.28	2.449	0.294
18. Our procedures and systems are good at preventing errors from happening	69.03	79.75	76.21	1.757	0.415
10. It is just by chance that more serious mistakes don’t happen around here	71.61	71.08	79.61	1.513	0.469
17. We have patient safety problems in this unit	72.18	70.70	79.32	1.324	0.516
**D3: Supervisor/Manager expectations and actions promoting patient safety**	71.45	76.41	76.14	0.420	0.798
19. My supervisor/manager expresses satisfaction when we try to avoid patient safety risks	71.04	87.39	69.09	5.190	0.075
20. My supervisor/manager seriously considers staff suggestions for improving patient safety	71.74	83.83	70.83	2.702	0.259
21. When work pressure increases, my supervisor/manager wants us to work faster, even though patient safety may be put at risk	70.34	65.18	84.83	6.196	**0.045**
22. My supervisor/manager overlooks patient safety problems that happen over and over	75.53	60.97	82.69	6.392	**0.041**
**D4: Organizational learning/continuous improvement**	70.48	81.50	73.63	1.574	0.455
6. We have activities to improve patient safety	72.43	82.29	71.21	2.043	0.360
9. When a failure in patient care is detected, appropriate measures are taken to prevent it from happening again	70.65	77.54	76.15	0.825	0.662
13. After we make changes to improve patient safety, we evaluate their effectiveness	74.31	79.96	70.98	1.241	0.538
**D5: Teamwork in the unit/service**	68.21	67.67	85.20	6.109	**0.047**
1. Staff support each other	69.28	70.00	82.59	4.402	0.111
3. When a lot of work needs to be done quickly, we work together as a team to get the work done	70.00	68.79	82.71	4.327	0.115
4. In this unit we all treat each other with respect	75.94	67.89	77.59	1.850	0.397
11. When someone is overloaded with work, they often find help from colleagues	70.76	72.71	79.39	1.883	0.390
**D6: Communication openness**	78.45	81.54	65.91	4.090	0.129
35. Staff will freely speak up if they see something that may negatively affect patient care	77.87	85.17	64.01	6.873	**0.032**
37. Staff feels free to question the decisions or actions of those with more authority	78.72	79.59	66.97	3.297	0.192
39. Staff are afraid to ask questions when something do not seem right	71.73	69.16	80.79	2.246	0.325
**D7: Feedback and communication about error**	75.18	88.99	64.02	8.252	**0.016**
34. When we report an incident, we are informed about what kind of action has been taken	73.53	82.70	69.88	2.469	0.291
36. We are informed of errors occurring in this service/unit.	78.37	81.49	66.03	4.477	0.107
38. In my service/unit we discussed how to prevent errors.	74.58	91.67	62.77	12.079	**0.002**
**D8: Non-punitive response to errors**	69.24	69.00	83.30	3.942	0.139
8. Staff feel like their mistakes are held against them	75.47	58.99	84.09	8.998	**0.011**
12. When a fault is detected, before looking for the cause, they look for a “culprit”	68.69	73.62	80.70	2.455	0.293
16. When a mistake is made, staff fear that it will be noted on their record	68.70	75.96	79.10	2.187	0.335
**D9: Staffing**	60.60	76.87	86.29	10.204	**0.006**
2. There are enough staff to cope with the workload	74.79	69.58	77.56	0.934	0.627
5. Sometimes the best patient care cannot be provided because the working day is exhausting	64.98	79.71	80.14	5.200	0.074
7. We use more agency/temporary staff than is best for patient care	66.68	76.17	80.91	3.468	0.117
14. We work under pressure to do too many things too quickly	63.91	73.51	85.38	9.595	**0.008**
**D10: Hospital management support for patient safety**	65.88	86.55	74.63	5.480	0.065
23. Hospital management provides a work climate that promotes patient safety	74.32	79.39	71.35	0.966	0.617
30. The actions of hospital management show that patient safety is a top priority	68.65	85.84	72.45	4.319	0.115
31. Hospital management seems interested in patient safety only after an adverse event happens	60.06	83.28	82.47	11.028	**0.004**
**D11: Inter-unit teamwork**	68.92	66.08	85.60	6.245	**0.044**
26. There is good cooperation among hospital units that need to work together	76.70	62.54	80.49	4.989	0.830
32. Hospital units work well together to provide the best care for patients	82.99	53.46	80.59	17.054	**0.000**
24. Hospital units do not coordinate well with each other	73.60	79.32	72.10	0.766	0.678
28. It is often unpleasant to work with staff from other hospital units	77.23	68.82	75.72	1.028	0.598
**D12: Problems with shift changes and transitions between units**	60.18	79.09	85.20	9.969	**0.007**
25. Patient information is partly lost when patients are transferred from one unit/service to another	69.74	72.74	80.29	2.066	0.356
27. Important patient care information is often lost during shift changes	68.99	67.14	84.80	6.314	**0.043**
29. Problems often occur in the exchange of information across hospital units	70.05	72.32	80.28	1.934	0.390
33. Shift changes are problematic for patients in this hospital	71.06	66.67	83.11	4.422	0.110

Bold numbers are statistical significance (*p* < 0.05).

**Table 4:** Adverse events identified.

Adverse Events	Total Cases	Total Prevalence *	Cases in Medical Units	Prevalence in Medical Units	Cases in Surgical Units	Prevalence in Surgical Units	Cases in Combined Units	Prevalence in Surgical Units
Phlebitis	100	3.312	36	5.042	31	4.341	33	4.621
Extravasations	81	2.683	30	4.201	30	4.201	21	2.941
Pressure ulcers	20	0.662	7	0.980	9	1.260	4	0.560
Accidental falls	3	0.099	0	-	3	0.420	0	-
urinary tract infections	1	0.033	1	0.140	0	-	0	-
Bacteremia	4	0.132	3	0.421	1	0.140	0	-
Surgical site infection	3	0.099	0	-	1	0.140	2	0.282
Other	2	0.066	2	0.280	0	-	0	-
Total	214		79		75		60	

* Total number of inpatients presenting an AE during the period under study/total number of patients in that period × 100.

## Data Availability

Not applicable.

## Appendix A

Patient Safety Questionnaire. Spanish version of the Hospital Survey on Patient Safety. Agency for Health Care Research and Quality (AHRQ): https://drive.google.com/drive/folders/1nNKD09L9uEbspF0Ns6CSwsa2-oTMTlNd?usp=sharing (accessed on 1 February 2022).

## Appendix B

Data collection form: https://drive.google.com/drive/folders/1RnVu0bMevbTx2rlUdsHGeVG53ko4gN7p?usp=sharing (accessed on 1 February 2022).
